# A Practical Model of Quartz Crystal Microbalance in Actual Applications

**DOI:** 10.3390/s17081785

**Published:** 2017-08-03

**Authors:** Xianhe Huang, Qingsong Bai, Jianguo Hu, Dong Hou

**Affiliations:** School of Automation Engineering, University of Electronic Science and Technology of China, Chengdu 611731, China; baiqingsong@std.uestc.edu.cn (Q.B.); hujianguo@std.uestc.edu.cn (J.H.); houdong@uestc.edu.cn (D.H.)

**Keywords:** quartz crystal microbalance (QCM), mass sensitivity function, equivalent mass sensitivity

## Abstract

A practical model of quartz crystal microbalance (QCM) is presented, which considers both the Gaussian distribution characteristic of mass sensitivity and the influence of electrodes on the mass sensitivity. The equivalent mass sensitivity of 5 MHz and 10 MHz AT-cut QCMs with different sized electrodes were calculated according to this practical model. The equivalent mass sensitivity of this practical model is different from the Sauerbrey’s mass sensitivity, and the error between them increases sharply as the electrode radius decreases. A series of experiments which plate rigid gold film onto QCMs were carried out and the experimental results proved this practical model is more valid and correct rather than the classical Sauerbrey equation. The practical model based on the equivalent mass sensitivity is convenient and accurate in actual measurements.

## 1. Introduction

The quartz crystal microbalance, comprised of a thin vibrating quartz wafer sandwiched between two metal excitation electrodes, has been used to determine interfacial mass changes through the mass dependence of the QCM resonant frequency. Over the last few decades, as a high-sensitive sensor, QCMs have been vigorously investigated in various fields [1,2,3,4], particularly in chemistry [5,6,7,8] and biomedical fields [9,10,11,12,13].

Sauerbrey put forward the famous Sauerbrey equation which describes the mass-frequency relationship at the QCM surface [14]

(1) $$\Delta m=-C_{QCM}\times \Delta f,$$

where Δm and Δf are mass change and frequency shift, respectively, and $C_{QCM}$ is the mass sensitivity constant which has a value of 17.7 ng·cm^−2^·Hz^−1^ for 5 MHz AT-cut QCMs.

In the past couple of decades, the Sauerbrey equation was the theoretical basis of using QCM to measurement in gas phase. Based on the Sauerbrey equation and other models [15,16,17,18,19], the QCM has been commonly used to detect a variety of nanoscale target analytes in liquid and gas environments due to advantages including good surface selectivity, simplicity of operation, real-time output, label-free analysis, and so on [20].

However, we should pay attention to the fact that the unit of the mass sensitivity constant $C_{QCM}$ is ng·cm^−2^·Hz^−1^, that is, for a 5 MHz AT-cut QCM, the resonant frequency will descend 1 Hz after 17.7 ng rigid film has been uniformly coated on 1 square centimeter surface area, or it means that the mass sensitivity within 1 square centimeter sensing area is about 5.65 × 10^10^ Hz/kg (the reciprocal of $C_{QCM}$). But in fact, the mass sensitivity of QCM is distributed as a Gaussian curve in the radial direction because of the energy trap effect of quartz crystal resonator, rather than having the same mass sensitivity in whole surface. Both theory and experiments proved that the mass sensitivity distribution of the QCMs coated with “m-m” type of electrodes is approximate Gaussian curves [21,22,23,24]. Besides, in order to ensure normal oscillation of QCMs, it is necessary to coat a layer of metal electrode on the both sides of quartz crystal wafer. The mass sensitivity function of QCMs may differ greatly depending on the material, shape, thickness, and size of the metal electrodes [21].

It is notable that the mass sensitivity constant $C_{QCM}$ in Sauerbrey equation is only related to the material constants of quartz crystal, but does not take both the Gaussian distribution characteristic of mass sensitivity and the influence of metal electrodes into consideration. Therefore, using the Sauerbrey equation unrestrictedly to calculate the mass change at QCM surface could bring about errors.

## 2. Theory

To overcome the above disadvantages of the classical Sauerbrey equation, this practical model is established on the basis of mass sensitivity function of the Sauerbrey equation. The mass sensitivity distribution for QCM devices is defined by [25,26,27]

(2) $$S_{f}\left(r,\theta \right)=\frac{{\left|A\left(r,\theta \right)\right|}^{2}}{2\pi \int_{0}^{\infty }r{\left|A\left(r,\theta \right)\right|}^{2}dr}\cdot C_{f},$$

where $S_{f}\left(r,\theta \right)$ is mass sensitivity function with a unit of (Hz/kg), $C_{f}$ is the Sauerbrey’s sensitivity constant with a value of 1.78 × 10^11^ Hz·cm^2^/kg [23], A(r,θ) is the particle displacement amplitude function and r is the distance from the center. In a QCM device, the particle displacement amplitude is invariant with the angular direction θ [21].

The particle displacement amplitude function A(r) in Equation (2) is solution of the following Bessel equation [23]

(3) $$r^{2}\frac{\partial^{2}A}{\partial r^{2}}+r\frac{\partial A}{\partial r}+\frac{k_{i}^{2}r^{2}}{N}A=0,$$

where N = 2.0443 according to the material constants of the AT-cut quartz crystal, and $k_{i}^{2}=\left(\omega^{2}-\omega_{i}^{2}\right)/c^{2}$, where i = *E,P,U* (*E*, *P*, and *U* represent the full electrode region, partial electrode region, and non-electrode region, respectively), $c=\sqrt{c_{66}/\rho_{q}}$ is the acoustic wave velocity in the crystal (where $c_{66}$ is the elastic stiffness constant, $\rho_{q}$ is the density of the quartz), $\omega_{i}$ is cut-off frequency of full electrode region ($\omega_{E}$), partial electrode region ($\omega_{P}$), and non-electrode region ($\omega_{U}$), respectively.

**Figure 1:**
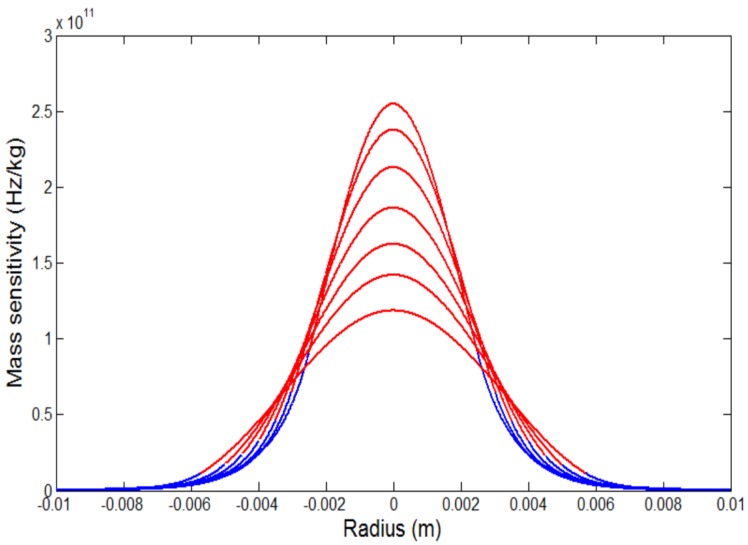
Mass-sensitivity distribution profiles for AT-cut 5 MHz QCMs with different-sized electrodes. From top to bottom in the figure the radiuses of the electrodes are, in order, 2.5, 3, 3.5, 4, 4.5, 5, and 5.7 mm. Red line represents the electrode region and blue line represents the non-electrode region.

**Figure 2:**
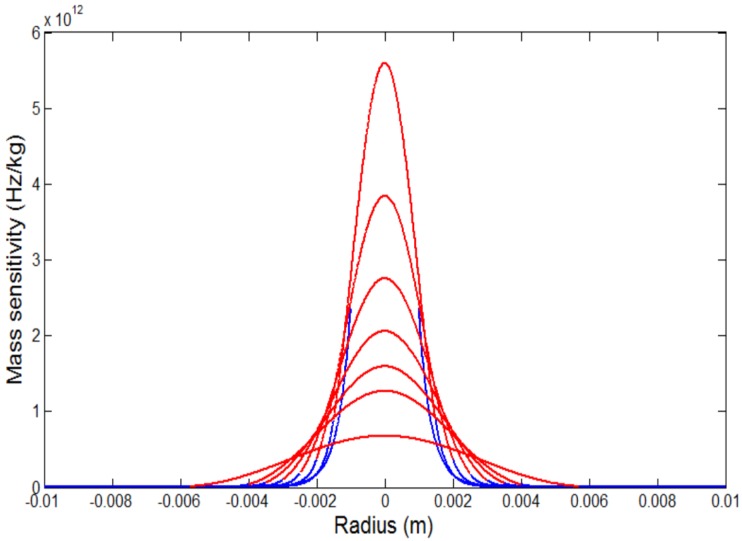
Mass-sensitivity distribution profiles for AT-cut 10 MHz QCMs with different-sized electrodes. From top to bottom in the figure the radiuses of the electrode are, in order, 1.5, 2, 2.5, 3, 3.5, 4, and 5.7 mm. Red line represents the electrode region and blue line represents the non-electrode region.

The mass sensitivity function $S_{f}\left(r\right)$ could be calculated according to the above mentioned analysis. Take 5 MHz QCMs and 10 MHz QCMs for examples, the profiles of mass-sensitivity distribution of 5 MHz QCMs and 10 MHz QCMs with different-sized electrodes can be obtained as shown in Figure 1 and Figure 2. The thickness of 5 MHz and 10 MHz quartz crystal wafers are about 0.333 mm and 0.167 mm, respectively, the thickness of the gold electrodes is 1000 Å. For each QCM, the highest sensitivity point appears at the center of the electrode (r = 0), and then decreases exponentially as the distance from the center (r) increases. Therefore, the mass sensitivity of QCMs differ greatly depending on the size of the electrodes and the distance from the center. Furthermore, smaller electrode will yield steeper mass sensitivity distribution curve while acquire higher absolute mass sensitivity.

As Figure 1 and Figure 2 shows, in view of the Gaussian features of the mass sensitivity distribution of QCMs, it will be more reasonable to take the integral of the mass sensitivity to indicate the equivalent sensitivity of a particular sensing area rather than a fixed constant $C_{QCM}$ in classical Sauerbrey equation. Therefore, the practical model of the Sauerbrey equation could be obtained as

(4) $$\Delta f=-\frac{\Delta m}{\pi r_{d}^{2}}\int_{0}^{r_{d}}2\pi rS_{f}\left(r\right)dr,$$

where $r_{d}$ is the radius of the particular circular region where mass loading attached on. Therefore, the fixed constant $C_{QCM}$ in the Sauerbrey equation should be replaced by an equivalent sensitivity $C_{QCM}^{*}$ (the unit of it is Hz·kg^−1^), that is, the practical model of Sauerbrey equation could also be expressed as

(5) $$\Delta f=-C_{QCM}^{*}\times \Delta m\;\left(\mathrm{where}\;C_{QCM}^{*}=\int_{0}^{r_{d}}2\pi rS_{f}\left(r\right)dr/\left(\pi r_{d}^{2}\right)\right),$$

**Figure 3:**
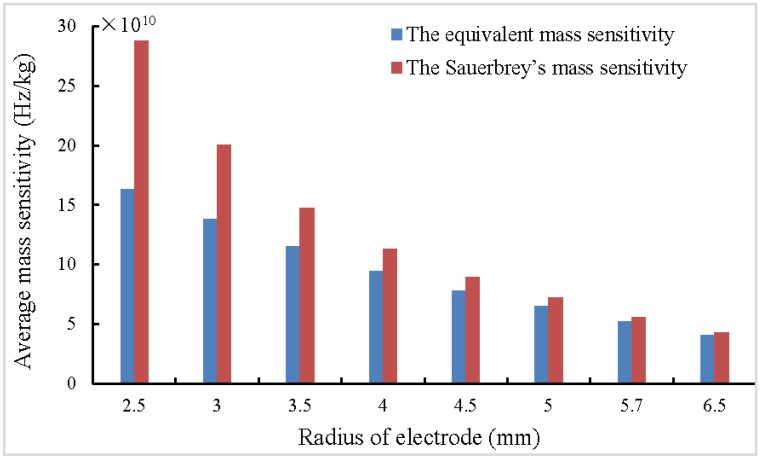
The equivalent mass sensitivities and the Sauerbrey’s mass sensitivity within electrode area of 5 MHz QCMs with different-sized electrodes.

**Figure 4:**
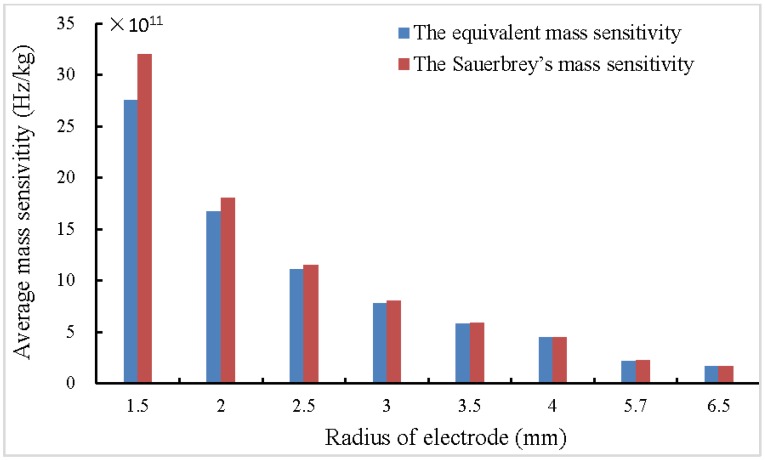
The equivalent mass sensitivities and the Sauerbrey’s mass sensitivity within electrode area of 10 MHz QCMs with different-sized electrodes.

According to the Sauerbrey equation, $\Delta f=-\Delta m/\left(\pi r^{2}C_{QCM}\right)$ the Sauerbrey’s mass sensitivity within loading area equals $1/\left(\pi r^{2}C_{QCM}\right)$, which is only related to the radius of added mass and the material constants of quartz crystal, but does not take the influence of metal electrodes into consideration. As Figure 3 and Figure 4 shows, the equivalent mass sensitivities $C_{QCM}^{*}$ within electrode area of 5 MHz and 10 MHz QCMs with different-sized electrodes were calculated according to Equation (5). The equivalent mass sensitivity and the Sauerbrey’s mass sensitivity both increases as the electrode radius decreases. The errors between them decrease as the electrode radius increases, and becomes small when the radius roughly equals to 5.7 mm (the electrode area roughly equals to 1 square centimeter). Moreover, higher resonant frequency results in higher mass sensitivity, therefore, enhancing the resonant frequency of QCM is a convenient and effective method to obtain higher mass sensitivity. On the other hand, because of the energy trap effect, smaller electrode is necessary to constitute a higher frequency QCM, which lets the Sauerbrey equation results in larger errors.

Through the analysis above, considering that the radial distribution of mass sensitivity strongly depends on the distance from the center and the size of electrodes, it is inaccurate to use Sauerbrey’s mass sensitivity to describe the mass-frequency relationship, and this practical model should be adopted to replace the classical Sauerbrey equation in practical applications.

## 3. Experimental

It is hard to directly measure the mass sensitivity of QCMs, for example, in reported research, Martin et al. [17] obtained the normalized mass sensitivity and Takayoshi Kawasaki et al. [28] obtained the relative values of mass sensitivity, but none of them measured the absolute sensitivity values directly. In other literature [25,26,27], some verified experiment were carried out and some trends could be observed, but the accuracy of these experiments are not high enough to validate its presented model.

**Figure 5:**
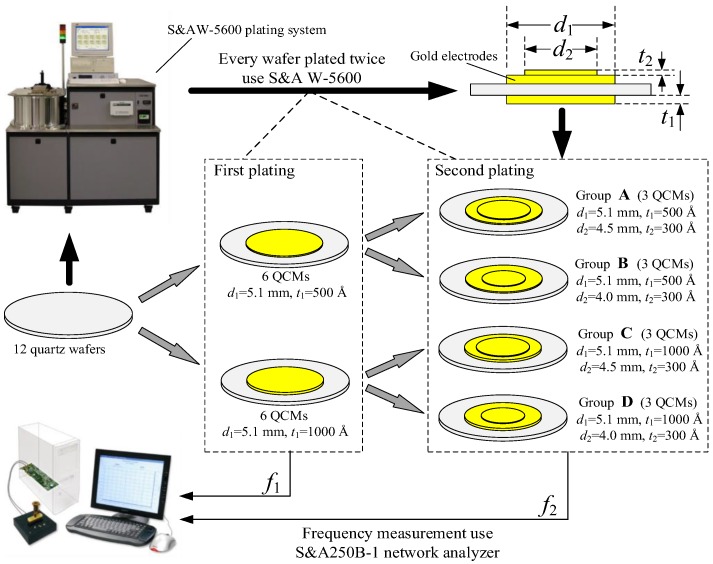
Schematic diagram of the experimental set-up.

A series of QCM experiments which plate rigid gold film on QCMs were performed to indirectly verify the validity and correctness of this practical model. Experimental environment was selected in a class 10,000 ultra-clean room of Wintron Electrionic Co., Ltd. (Zhengzhou, China). The ambient temperature in the ultra-clean room is maintained at 23 °C. Twelve ‘plano-plano’ quartz wafers with a fundamental frequency of 10 MHz and a diameter of 8.7 mm were used in the experiment. Figure 5 is a schematic of the experimental set-up. To investigate the correctness of the practical model, these QCMs were divided into four groups according to different diameters and thicknesses of electrodes and plated thin films.

In the first plating process, groups A and B were plated gold electrodes with a diameter of 5.1 mm and a thickness of 500 Å onto both side; groups C and D were plated gold electrodes with a diameter of 5.1 mm and a thickness of 1000 Å onto both sides, and their resonant frequencies were measured and recorded as $f_{1}$. In the second plating process, groups A and C were plated gold electrodes with a diameter of 4.5 mm and a thickness of 300 Å onto the upper surface; groups B and D were plated gold electrodes with a diameter of 4.0 mm and a thickness of 300 Å onto the upper surface, and their resonant frequency were measured and recorded as $f_{2}$. $\Delta f_{e}=f_{1}-f_{2}$ is the frequency shift caused by thin gold film which was been plated in the second plating process. Note that these quartz wafers and electrodes are circular, so the angular direction θ has not been considered.

The equipment used in plating process is the S&A W-5600 BASE PLATING SYSTEM (Saunders & Associates, LLC. Phoenix, AZ, USA). The coating thickness is set by the equipment program.

## 4. Discussion

The frequency of all the 12 QCMs (four groups) were measured use the S&A250B-1 network analyzer (Saunders & Associates, LLC. Phoenix, AZ, USA), and the results are shown in Table 1. $\bar{\Delta f_{e}}$ and δ are the average value and the standard deviation of $\Delta f_{e}$, respectively. Δm is the mass change caused by the second plating. $\Delta f_{t}$ and $E_{t}$ are the theoretical frequency change according to Equation (5) and the error between $\Delta f_{t}$ and $\bar{\Delta f_{e}}$, respectively. $\Delta f_{s}$ and $E_{s}$ are the theoretical frequency change according to Sauerbrey equation and the error between $\Delta f_{s}$ and $\bar{\Delta f_{e}}$, respectively.

The best standard deviation (44.18 Hz) in these experiments is obtained in the group B, and the maximum standard deviation (238.84 Hz) is obtained in group A. This low standard deviation shows the high stability of the experimental system and the environment.

As Table 1 shows that the electrode radius and thickness did have influence on QCM’s frequency–mass relationship, but the Sauerbrey equation did not consider it. Regarding group A and group C, for a 10 MHz QCM with an electrode diameter of 5.1 mm attached by a mass loading with a diameter of 4.5 mm, the Sauerbrey’s mass sensitivity within the loading area is not much different from the equivalent mass sensitivity, therefore the errors between the theoretical frequency change $\Delta f_{s}$ according to Sauerbrey equation and the experimental results are small.

Nevertheless, for groups B and D, as Figure 4 shows, the deviation between the Sauerbrey’s mass sensitivity and the equivalent mass sensitivity of a QCM with a 4 mm electrode diameter is just about 7.7%, the theoretical results according to the Sauerbrey equation are far different from the experimental results with errors of about 50%. In this study, the QCM is the quartz resonator with the first plating, and the attached mass is coated by the second plating. For groups B and D, the attached mass is much centered than groups A and C, the equivalent mass sensitivity is the integral average of $S_{f}\left(r\right)$ within the second plating area with a diameter of 4.0 mm, and the diameter of the electrodes is 5.1 mm instead of 4 mm. This equivalent mass sensitivity is much smaller than the mass sensitivity used in the classical Sauerbrey equation within the 4 mm diameter sensing area, then the error reached about 50% in groups B and D. Therefore, in this case, with this presented model, an error of about 50% of the final measured results could be avoided.

On the other hand, the theoretical results $\Delta f_{t}$ according this practical model of all the four groups agree well with the experimental results, all the errors are less than 4%.

## 5. Conclusions

The above experiment shows that the Sauerbrey’s mass sensitivity cannot accurately reflect the frequency vs. mass loading relationship of QCM, especially for these cases which with a small electrode or with a large electrode loaded by a small loading. A much more accurate result could be obtained by using the equivalent mass sensitivity in this practical model. By using the integral of sensitivity function to indicate the frequency–mass relationship, the practical model presented in this paper which considered both the Gaussian distribution characteristic of mass sensitivity and the influence of the electrodes is more accurate than the Sauerbrey equation. Both theoretical analysis and experimental results proved the validity and correctness of this practical model. In view of the widespread use of the Sauerbrey equation, the new practical model should be widely used because of its higher accuracy.

## Figures and Tables

**Table 1:** Experimental results and theoretic values.

	A			B			C			D		
$f_{1}$ (Hz)	10,006,391	10,006,821	10,004,123	10,008,944	10,003,835	10,005,320	9,958,942	9,961,677	9,961,850	9,957,356	9,958,215	9,956,112
$f_{2}$ (Hz)	9,993,685	9,994,674	9,991,846	9,998,311	9,993,262	9,994,795	9,946,278	9,949,200	9,949,227	9,945,483	9,946,365	9,944,688
$\Delta f_{e}$ (Hz)	12,706	12,147	12,277	10,633	10,573	10,525	12,664	12,477	12,623	11,873	11,850	11,424
$\bar{\Delta f_{e}}$ (Hz)	12,377			10,577			12,588			11,716		
δ (Hz)	238.84			44.18			80.25			206.45		
Δm (kg)	9.22 × 10^−9^			7.28 × 10^−9^			9.22 × 10^−9^			7.28 × 10^−9^		
$C_{QCM}^{*}$ (Hz/kg)	1.29 × 10^12^			1.50 × 10^12^			1.35 × 10^12^			1.59 × 10^12^		
$\Delta f_{t}$ (Hz)	11,891			10,913			12,444			11,581		
$E_{t}$	−3.93%			+3.35%			−1.14%			−1.15%		
$\Delta f_{s}$ (Hz)	13,120			16,596			13,120			16,596		
$E_{s}$	+6.0%			+56.9%			+4.2%			+41.7%		
